# Late Cystic Metastasis of an Ovarian Granulosa Cell Tumor to the Liver

**DOI:** 10.7759/cureus.18051

**Published:** 2021-09-17

**Authors:** Ammar Aleter, Parag S Mahajan, Mahir A Petkar, Hatem A Abdulmajeed, Hatem Khalaf, Walid Elmoghazy, Ahmed Elaffandi

**Affiliations:** 1 Department of Surgery, Hamad Medical Corporation, Doha, QAT; 2 Department of Clinical Imaging, Hamad Medical Corporation, Doha, QAT; 3 Department of Laboratory Medicine & Pathology, Hamad Medical Corporation, Doha, QAT; 4 College of Medicine, Qatar University, Doha, QAT

**Keywords:** sex cord stromal tumors, granulosa cell tumor, liver metastasis, late recurrence, partial hepatectomy

## Abstract

Granulosa cell tumor (GCT) is a unique form of sex cord tumor that is mostly unilateral and of low-grade malignancy. Most GCT recurrence is with pelvic or peritoneal dissemination. Liver metastasis is rarely reported. This study reports a rare case of GCT with liver metastasis nine years post initial presentation. We also discuss surgical intervention, radiological findings, histology, treatment approaches, and review of similar reported cases.

## Introduction

The World Health Organization’s histological classification of ovarian tumors in 2014 defined granulosa cell tumor (GCT) as a subtype of sex cord-stromal tumor. These tumors are low-grade malignant tumors, which consist of pure sex cord tumors and pure stromal tumors [1]. The GCT is a rare type of tumor and is considered a pure stromal tumor, accounting for 2-5% of ovarian tumors and 70% of sex cord-stromal tumors [2]. It is divided into adult GCTs, which is the most common with an incidence of 95%, and juvenile GCTs [2]. GCT is typically treated by ovariectomy and has a good prognosis in general; nevertheless, late recurrence can be found. GCT metastasis and recurrence are often encountered with pelvic or peritoneal dissemination, whereas distant metastases to the lung, brain, and liver are less common [3]. Liver metastasis was noted only in 5-6% of the cases [3].

Ovarian GCT with liver metastasis is seldom documented, and liver metastasis can be easily misdiagnosed as a mucinous cystadenoma or primary liver cancer. For which, surgical removal may not be performed, leading to a deteriorative pathogenetic condition [4]. Resection of liver metastases for GCTs is frequently performed as a palliative rather than a therapeutic procedure; however, in many cases, it may improve the quality of life significantly [4].

This study reports with a literature review the case of a patient in whom primary GCT of the ovary was diagnosed more than nine years prior to liver metastatic resection.

Our case is unique, as it was radiologically evaluated by ultrasonography (US), computed tomography (CT) scan, magnetic resonance imaging (MRI) scan, and fluoro-deoxy glucose positron emission tomography (FDG-PET) CT scan, and the diagnosis of delayed hepatic metastatic recurrence was suspected preoperatively on MRI and PET-CT. The diagnosis of hepatic metastasis from GCT was strongly considered on the MRI scan due to the clinical history and some similarities in the appearance of the hepatic metastasis with the primary GCT of the ovary on the preoperative pelvic MRI done nine years ago. Also, the hepatic lesion showed low-level FDG uptake on PET-CT that is typical of ovarian GCTs.

## Case presentation

This patient is a 56-year-old lady who presented to the emergency department on November 27, 2020, regarding on and off severe epigastric pain radiating to her back for one month associated with nausea. The patient did not report any feeling of abdominal mass, and she did not have any weight or appetite loss during the past months. She is post-menopausal with no complaint of vaginal bleeding, discharge, or lower abdominal pain. There were no other gastrointestinal or urologic symptoms, and her systemic review was unremarkable. Her past surgical history is significant for an open surgical intervention done due to a right ovarian tumor diagnosed in March 2012. The surgical intervention was cytoreductive surgery, including total abdominal hysterectomy, bilateral salpingo-oophorectomy, ascites cytology, bilateral pelvic lymph node dissection, para-aortic lymph node sampling, and omental biopsy. Her final histopathology diagnosis came as an adult-type granulosa cell tumor of the right ovary stage III-A.

The patient received adjuvant chemotherapy starting from May up to September 2012 composed of bleomycin, etoposide, cisplatin (BEP) protocol for one cycle; however, the protocol was shifted to paclitaxel and carboplatin due to severe neutropenia and gastrointestinal discomfort. The patient received six cycles of the second protocol with no significant side effects. She was followed closely for five years in the clinic, up to 2017, by physical examinations and scheduled imaging with no signs of recurrence and was then lost to further follow-up. Her family history is positive for breast cancer in her cousin and lung cancer in her father, and she also had multiple comorbidities, including diabetes mellitus type two, gastritis, and benign thyroid nodules.

**Figure 1:**
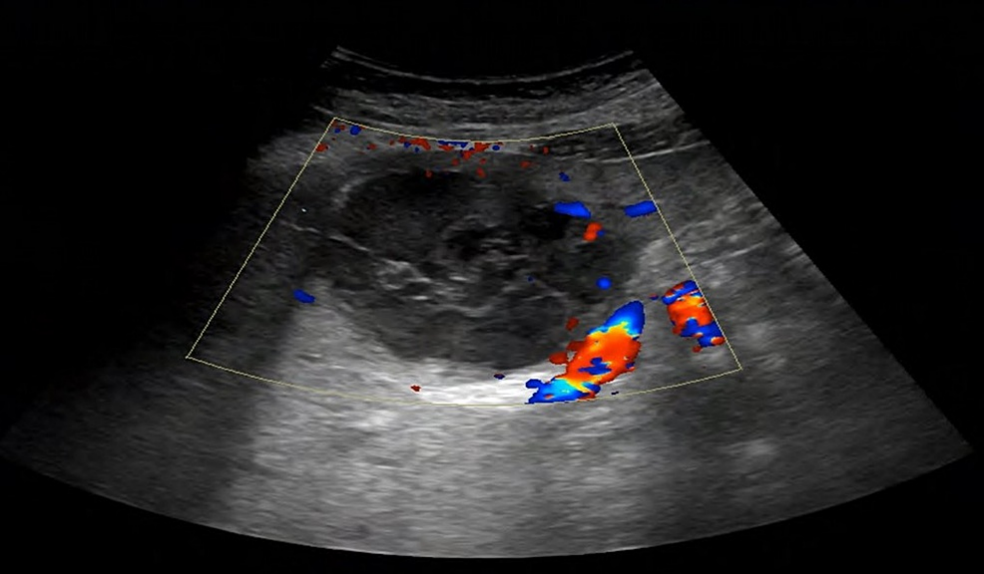
Liver ultrasound showing a complex cystic mass lesion with vascularity in the wall and the septae

**Figure 2:**
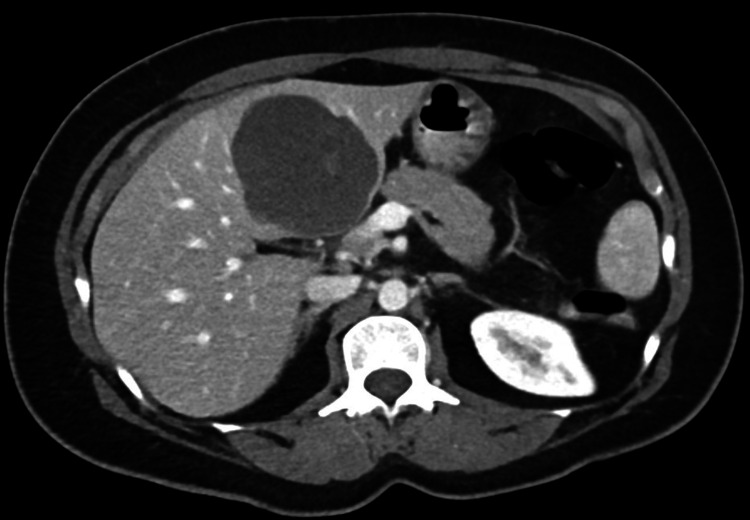
Post-contrast (venous phase) CT scan of the liver showing a complex cystic mass lesion in the left lobe of the liver with an enhancement of the wall and the septae

On examination in the emergency department in November 2020 (nine years later), the patient was found to have mild tenderness in the epigastric area, no guarding or rigidity, and no masses or hernias could be felt. All her laboratory results, including complete blood count (CBC), liver enzymes, hepatitis serology, tumor markers, such as AFP (alpha-fetoprotein), CA19-9 (carbohydrate antigen 19-9), CA 125 (cancer antigen 125), CA 15-3 (cancer antigen 15-3), and estradiol, were within the normal range. Anti-Mullerian hormone (AMH), which is considered currently by many authors as an efficient hormone for the diagnosis and follow-up of granulosa cell tumors, was within the normal range as well [5]. On the other hand, her inhibin-B value, which is another tumor marker, proved to be efficient in detecting both primary and recurrent disease was elevated (120 PG/ML) [6-7]. The US done at presentation showed an ill-defined, mixed, echogenic, predominantly hypoechoic focal lesion with posterior acoustic enhancement and irregular margins measuring 5.7 x 6.6 x 5.3 cm in the left lobe of the liver (Figure 1). No obvious vascularity was noted within the lesion with minimal peripheral and septal vascularity. Features suggested a complex cystic lesion. CT scan with intravenous (IV) contrast confirmed the US findings (Figure 2).

**Figure 3:**
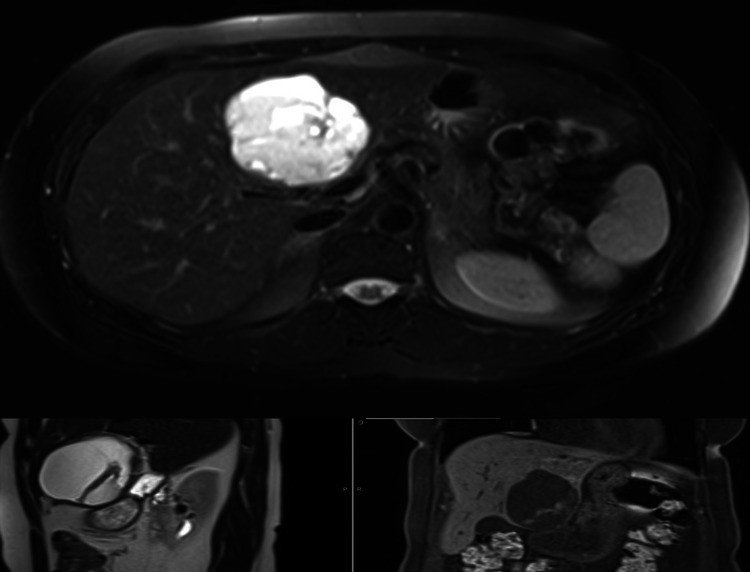
MRI of the liver shows a complex cystic mass lesion in the left lobe of the liver

**Figure 4:**
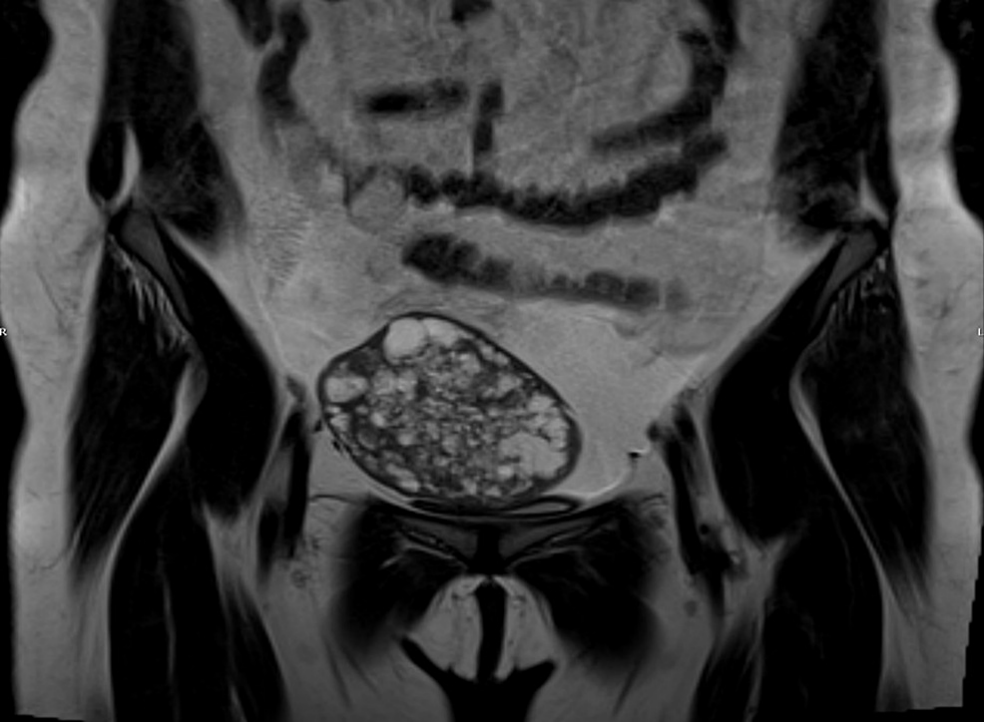
Coronal pre-operative MRI of pelvis done in 2012 shows a complex cystic mass lesion in the pelvis – primary granulosa cell tumor of the ovary

**Figure 5:**
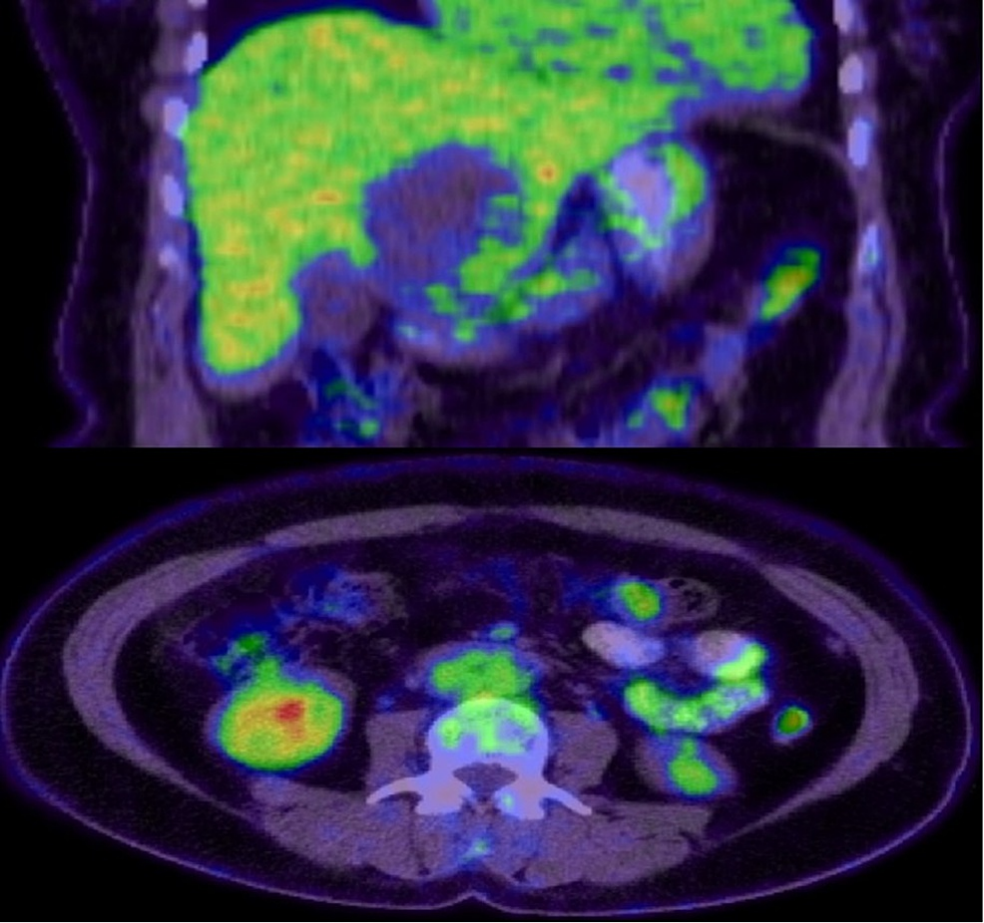
PET-CT showing mild radiotracer uptake by the left hepatic lobe complex cystic lesion (SUVmax 3.8) and the peritoneal nodule in the left side of the abdomen PET-CT: positron emission tomography-computed tomography

MRI done to further delineate the lesion showed a well-defined, well-encapsulated, rounded, multilocular, septated, and complex cystic lesion in the left lobe of the liver (segments III, IVb) (Figure 3). It measured approximately 7.9 x 6.8 x 7.8 cm. It was heterogeneously hyperintense on both T1 and T2-weighted images with hypointense capsule and septa on both sequences. It showed fluid-debris levels within likely, suggestive of chronic hemorrhage/highly proteinaceous content. There were enhancing nodular and papillary projections with diffusion restriction in the soft tissue nodules, suspicious of malignant transformation. The lesion was seen stretching the liver capsule, abutting the pyloric region of the stomach, the duodenal bulb, the junction between the head and body of the pancreas, and the main portal vein; otherwise, the liver was normal in signal intensity and enhancement pattern. In the arterial phase, there was no appreciable enhancement, and in the venous and delayed phases, it showed enhancement of the capsule and the internal septa and no appreciable enhancement of the rest of the lesion. There was mild hepatomegaly; otherwise, the rest of the liver was normal. Preoperative MRI of pelvis done in 2012 was available for comparison (Figure 4). Some features of the current hepatic lesion (complex cystic appearance, enhancing wall, papillary projections and multiple internal septations, and internal hemorrhage) were similar to those of the primary ovarian GCT. In view of the patient’s history, a primary diagnosis of hepatic metastatic recurrence of GCT was considered with a differential diagnosis of malignant transformation of a biliary cystadenoma. US-guided biopsy of the hepatic lesion was unsatisfactory. Hence, after discussion in a hepatobiliary multidisciplinary meeting, the decision was to proceed with a PET-CT scan, and it showed a low-level FDG uptake consistent with recurrence of the disease (Figure 5). It additionally showed a small peritoneal deposit.

**Figure 6:**
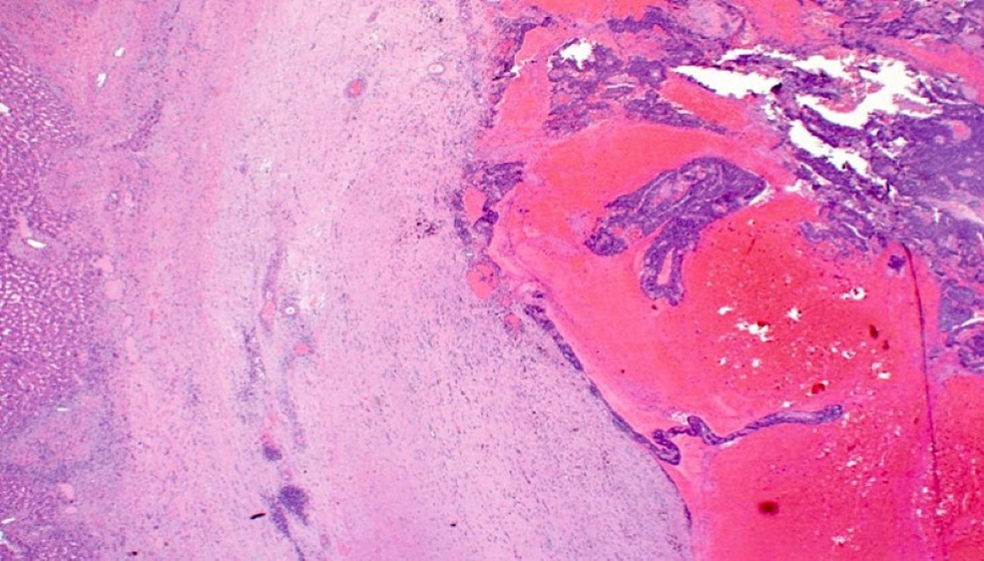
Low power view revealing a cystic tumor (right) with blood clots within the cyst and background liver parenchyma (left) (H and E x2)

**Figure 7:**
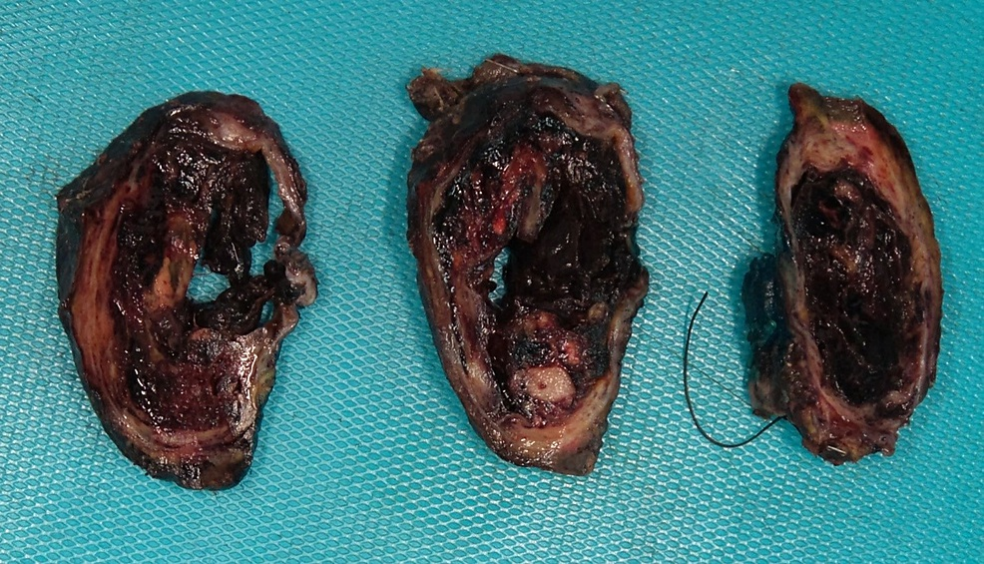
Macroscopic image of the cystic tumor, exhibiting marked hemorrhagic cystic degeneration

**Figure 8:**
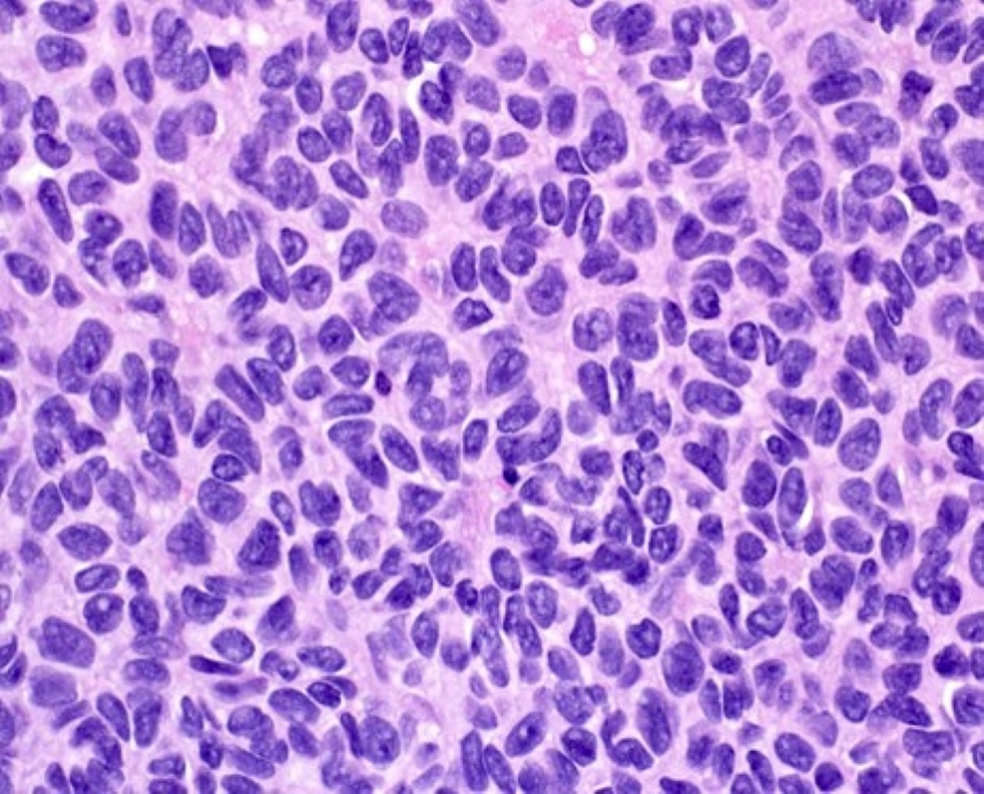
High power displaying the characteristic "coffee bean" nuclear appearance of granulosa cell tumor (H and E x20)

The patient was admitted for surgical intervention on the third of March 2021 to resect the liver metastasis, and surgery started with diagnostic laparoscopy to rule out disseminated metastatic disease. The lesion was cystic in texture, measuring around 6X7 cm, and it was occupying segments III and IVb of the left liver lobe. Another small peritoneal nodule measuring around 2X2 cm was found in the right hepatorenal recess. After ruling out a disseminated metastatic disease by diagnostic laparoscopy, we proceeded with the resection by an inverted T-shaped incision and opening the abdominal wall in layers. Adhesiolysis was done around the left liver lobe and then a modified left hepatectomy was done after marking our resection line, which was to the left of the middle hepatic vein to resect segments II, III, and IVb with preservation of segment IVa. The peritoneal nodule was also excised from the hepatorenal recess. The patient had a smooth recovery postoperatively and was discharged on the fifth day of surgery with no significant complications. Histopathological examination of the cystic liver lesion (7.2 x 5 x 4.8 cm) revealed a cystic neoplasm composed of tumor cells exhibiting the characteristic features of granulosa cell tumor (Figures 6-7). The tumor nuclei had prominent grooves, imparting the classical “coffee bean” appearance (Figure 8). Whilst Call-Exner bodies are usually seen in the microfollicular architectural pattern of granulosa cell tumor, this feature was not noted in our case, as it had a diffuse architectural pattern.

**Figure 9:**
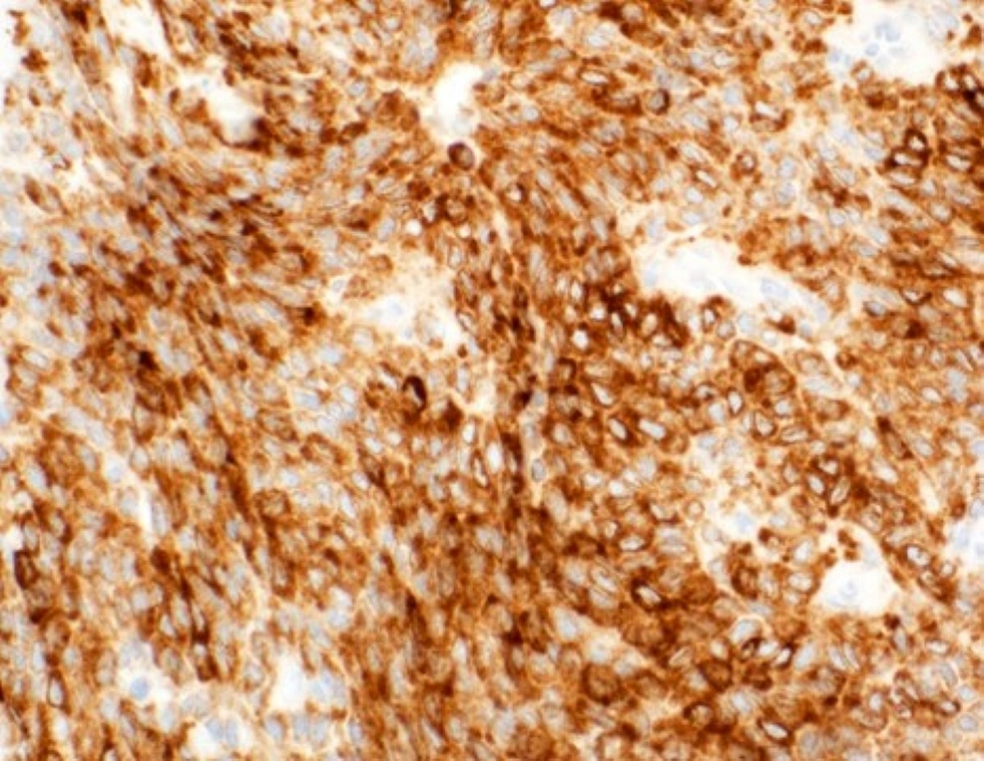
Strong diffuse inhibin positivity (x 10)

Immunohistochemically, the tumor was positive for inhibin (Figure 9), WT1 (Wilms' tumor suppressor gene 1), and calretinin (focal), confirming the histological diagnosis. Immunostains for cytokeratin CKAE1/AE3, CK7, and EMA were negative. Similar histological features were noted in the peritoneal nodule (2.2 x 1.5 cm), indicating another focus of metastatic granulosa cell tumor. Both metastatic foci were completely excised with adequate margins.

The patient has been following in the clinic for the past months with no new complaint. The gynecology multidisciplinary meeting plan is to follow the patient closely with AMH and inhibin, with no need for further hormonal or chemotherapy treatments.

## Discussion

Ovarian cancer has the fifth highest mortality rate of all female cancers, repre­senting 5‑6% of cancer‑related mortalities [8]. Overall, ovarian cancers originate from the epithelium surface in 85% of the cases while sex cord‑stromal tumors represent 2-5% only, with GCT being the most common [9]. GCT frequently presents in multiparous women and can occur at any age but generally presents during the early postmenopausal period with a median age between 46 and 50 years in most series [10].

The two main subtypes of GCTs are adult GCTs (AGCTs) and juvenile GCTs (JGCTs) with AGCT as the most common [11]. GCT has never been reported to be originating from the liver previously and even liver metastasis from ovarian GCT is very rare, with only 18 reported cases (including this case) that underwent hepatectomy since the first description by Garcia et al. in 1996 [12-13].

Table 1 shows the epidemiology and clinical characteristics of the ovarian GCTs with liver metastasis who underwent hepatectomy, and we can notice that most of the liver metastases were diagnosed years after the primary tumor, which is the same in our patient. This finding concurs with the current literature suggesting that ovarian GCTs are commonly low‑grade malignancies with a long, disease‑free interval owing to the indolent nature of the disease. However, most patients must have prolonged follow-up and be aware of the fact that this type of tumor is well-known to have late recurrences with an incidence of 25‑30% [20]. Hepatic metas­tases on the other hand are infrequently encountered, with an incidence of 5‑6% of all GCT recurrences [18].

**Table 1:** Literature review: epidemiology and clinical characteristics of ovarian GCT patients with liver metastasis who underwent a hepatectomy * Not applicable GCT: granulosa cell tumor

Author	Year of publication	No. of cases	Age at presentation for liver metastasis (years)	Symptoms at presentation	Other organs metastasis or invasion	Time from primary diagnosis to liver metastasis (months)	Time from liver metastasis to hepatectomy (months)
[13] Rodriguez Garcia et al.	1996	1	62	abdominal mass	none	72	60
[14]Crew et al.	2005	1	58	dyspnea	lung	144	120
[15]Lordan et al.	2007	1	64	recurrent chest infection, abdominal pain	lung, peritoneal dissemination	180	72
[4]Madhuri et al.	2010	3	N/A*	dyspnea Abdominal pain (2 patients)	peritoneal dissemination	204/ 108/ 72	32 /12 /0
[16]Chua et al.	2011	2	46/ 31	N/A	lung and peritoneal dissemination	144/ 72	0/ 172
[17]Andreou et al.	2012	5	N/A	N/A	N/A	N/A	N/A
[3]Fujita et al.	2015	1	43	abdominal distention and ascites	peritoneal dissemination	300	0
[18]Yu et al.	2015	1	62	abdominal pain	jejunal metastasis	324	0
[19]Antony et al.	2017	1	35	abdominal pain and ascites	right diaphragm	63	50
[12]Koganezawa et al.	2020	1	76	incidental	right diaphragm	264	96
Present case	2021	1	56	abdominal pain	peritoneal nodule	108	0

The longest recurrence-free survival period of granulosa cell tumor was reported by East et al., which was 40 years after the primary tumor, and occurred in the pelvis [21], while the longest recurrence-free survival period with hepatic metastasis was reported by Yu et al. and was 27 years post-primary tumor [22]. In our case, recurrence occurred nine years after treatment of the primary tumor.

The most common presenting symptom of ovarian GCT is vaginal bleeding (16.7-46%), followed by palpable abdominal mass (28.4%) and abdominal pain (17.6-44.3%) [23]. We can notice from Table 1 that most patients with ovarian GCT and liver metastasis were presenting with abdominal pain (33.3%) while the incidental discovery of the liver metastasis during follow-up for another pathology ensued in only one out of the 18 patients.

None of the patients were found to have a recurrence during their regular follow-up post-treatment of the primary tumor. This is related mainly to the fact that those patients have already been lost to follow-up due to tumor recurrence years after primary presentation with a recurrence-free survival mean of 158 months (13 years) (Table 1).

As for diagnosis, the most used tumor markers for GCT detection and recurrence are AMH and inhibin (mainly Inhibin B) [22]. In a partially prospective cohort study, Färkkilä et al. collected data of 123 patients with 10.5 years as a median follow-up to evaluate the efficiency of both AMH and inhibin B as tumor markers for GCT. AMH has been shown to be a specific circulating marker for GCT. Its diagnostic performance appears to be effective for primary and recurrent GCT with 92% sensitivity and 81% specificity [6].

With respect to inhibin as a tumor marker, there is two main subtypes (inhibin A and B). Inhibin B is the main form secreted by granulosa cell tumors, and it is more accurate in reflecting disease status than inhibin A. Therefore, measurement of serum inhibin B level is preferred for the follow-up of GCT [22]. This finding is particularly accurate in our case, as inhibin A was normal in this patient after recurrence while the inhibin B value was elevated. Serum estrogen and CA 125 concentrations have been used to detect primary tumor and recurrences; however, a consistent correlation could not be confirmed between their levels and tumor activity [10,24].

CT scan was the main modality for follow-up and detection of recurrence preoperatively as we can notice in Table 2. Although Koganezawa et al. performed PET-CT during the evaluation of his patient, no significant FDG uptake could be found in the hepatic tumor or other parts of the body. Hence, the tumor was suspected to be a liver hemangiosarcoma [12]. To our knowledge, this is the first report of preoperative PET-CT diagnosis of hepatic metastatic recurrence of ovarian GCT. Also, ours is the first report showing comparative MRI features of the hepatic metastasis and the primary GCT.

**Table 2:** Literature review: diagnosis modality and surgical intervention type of ovarian GCT patients with liver metastasis * Not applicable; ** Cancer antigen 125; *** Alpha-fetoprotein; **** Anti-Mullerian hormone GCT: granulosa cell tumor

Author	Follow-up tumor markers	Imaging modality used	Type of liver resection	Combined resection of other organs	recurrence
[13]Rodriguez Garcia et al.	N/A*	US, CT scan	Right hepatectomy	No	No
[14] Crew et al.	Estradiol, inhibin	CT scan	Partial hepatectomy	Yes	Yes
[15]Lordan et al.	N/A	CT scan, MRI	Extended right hepatectomy	Yes	Yes
[4]Madhuri et al.	N/A	N/A	Partial hepatectomy	Yes	No
[16]Chua et al.	N/A	N/A	Partial, right hepatectomy	Yes	1 Yes 1 / No
[17]Andreou et al.	N/A	N/A	N/A	N/A	N/A
[3]Fujita et al.	Estradiol, CA 125**	US, CT scan, MRI	Right hepatectomy	Yes	Yes
[18]Yu et al.	AFP***	CT scan	Central bisectionectomy	No	No
[19]Antony et al.	CA 125, AFP	CT scan	Partial, right hepatectomy	Yes	No
[12] Koganezawa et al.	AFP	CT scan, MRI, PET-CT	Right trisectionectomy	No	No
Present case	Estradiol, inhibin, AMH****, AFP	US, CT scan, MRI, PET-CT (could not appreciate recurrence)	Left hepatectomy	Yes	Under follow up

Concerning treatment, primary surgery is the standard treatment for both types of GCTs. It is generally curative in most patients due to the early stage and unilateral involvement [22]. Typically, surgery should comprise of hysterectomy and bilateral salpingo-oophorectomy. Lymph node dissection is of limited value, mainly in early-stage disease [22]. In advanced disease patients (stages II-IV), cytoreductive surgery should be done to resect as much as possible tumor load and metastases [25]. Many authors reported that removal of the remaining disease can significantly enhance a patient’s postoperative quality of life and recurrence-free survival [25]. The role of adjuvant chemo and radiotherapy is mainly for patients with advanced or recurrent disease. In early-stage disease, their efficiency is still unclear as its utilization did not affect disease-free survival [26].

In our patient left hepatectomy was done as liver metastasis was expected to be the only form of GCT recurrence. No further adjuvant therapy was offered for the patient after treating this recurrence and so far, no recurrence of liver metastasis was observed.

## Conclusions

GCT recurrence as liver metastasis is an extremely rare finding that can be encountered late post-resection of the primary tumor. Inhibin B and AMH are the most accurate tumor markers for recurrence detection. Variable imaging modalities could be used for diagnosis, including MRI and PET-CT. Surgical resection for advanced disease and disease recurrence to remove the remaining disease can enhance the patient’s postoperative quality of life and recurrence-free survival.
